# Supplementary material for: Complexome profiling of the Chlamydomonas psb28 mutant reveals TEF5 as an early PSII assembly factor
Source: Plant Cell. 2025 Mar 17;37(6):koaf055. doi: 10.1093/plcell/koaf055 (PMC12164590; doi:10.1093/plcell/koaf055)
Supplement: koaf055_Supplementary_Data [file koaf055_supplementary_data.zip › psb28_interactive.zip!/plots/intensity/qualityPlots/peptides/COX13_Cre12.g537450.t1.2.html]

### Cre12.g537450.t1.2

Trivial name: COX13  
SeqCoverage [median]: 36.80  
Mass: 13.97 kDa  
  
 

| Sequence | MissedCleavage | Mass [Da] | wtIntensity | muIntensity | Score |
| --- | --- | --- | --- | --- | --- |
| ASTTAGETIDK | 0 | 1092.53 | 2312.23 | 769.58 | 111.06 |
| ASTTAGETIDKYWAPYFPKPAVTADEAK | 2 | 3027.49 | 807.76 | 0.00 | 30.14 |
| LPGMPWGQDGLFEGHPR | 0 | 1892.90 | 1994.73 | 4368.00 | 73.21 |
| PAVTADEAK | 0 | 900.46 | 1117.00 | 1575.10 | 131.06 |
| RLPGMPWGQDGLFEGHPR | 1 | 2049.00 | 1420.22 | 1280.41 | 56.22 |
|  |  |  | 7651.9 | 7993.1 |  |
